# *Zip4* (*Slc39a4*) Expression is Activated in Hepatocellular Carcinomas and Functions to Repress Apoptosis, Enhance Cell Cycle and Increase Migration

**DOI:** 10.1371/journal.pone.0013158

**Published:** 2010-10-04

**Authors:** Benjamin P. Weaver, Yuxia Zhang, Stephen Hiscox, Grace L. Guo, Udayan Apte, Kathryn M. Taylor, Christian T. Sheline, Li Wang, Glen K. Andrews

**Affiliations:** 1 Department of Biochemistry and Molecular Biology, University of Kansas Medical Center, Kansas City, Kansas, United States of America; 2 Departments of Medicine and Oncological Sciences, Huntsman Cancer Institute, University of Utah School of Medicine, Salt Lake City, Utah, United States of America; 3 Tenovus Centre for Cancer Research, Welsh School of Pharmacy, Cardiff University, Cardiff, United Kingdom; 4 Department of Pharmacology, Toxicology and Therapeutics, University of Kansas Medical Center, Kansas City, Kansas, United States of America; 5 Neuroscience Center of Excellence, Louisiana State University Health Science Center, New Orleans, Louisiana, United States of America; The University of Hong Kong, China

## Abstract

**Background:**

The zinc transporter ZIP4 (*Slc39a4*) is important for proper mammalian development and is an essential gene in mice. Recent studies suggest that this gene may also play a role in pancreatic cancer.

**Methods/Principal Findings:**

Herein, we present evidence that this essential zinc transporter is expressed in hepatocellular carcinomas. *Zip4* mRNA and protein were dramatically elevated in hepatocytes in the majority of human hepatocellular carcinomas relative to noncancerous surrounding tissues, as well as in hepatocytes in hepatocellular carcinomas occurring in *farnesoid X receptor*-knockout mice. Interestingly, meta-analysis of microarray data in the Geo and Oncomine databases suggests that *Zip4* mRNA may also be elevated in many types of cancer. Potential mechanisms of action of ZIP4 were examined in cultured cell lines. RNAi knockdown of *Zip4* in mouse Hepa cells significantly increased apoptosis and modestly slowed progression from G_0_/G_1_ to S phase when cells were released from hydroxyurea block into zinc-deficient medium. Cell migration assays revealed that RNAi knockdown of *Zip4* in Hepa cells depressed *in vitro* migration whereas forced over-expression in Hepa cells and MCF-7 cells enhanced *in vitro* migration.

**Conclusions:**

ZIP4 may play a role in the acquisition of zinc by hepatocellular carcinomas, and potentially many different cancerous cell-types, leading to repressed apoptosis, enhanced growth rate and enhanced invasive behavior.

## Introduction

Zinc is essential for the structure and function of literally hundreds of proteins [1]. Therefore, changes in zinc availability alter numerous cellular processes. Multiple genes have evolved to control the storage (*Metallothionein* genes), efflux (*Slc30a*; *Znt* genes) and uptake (*Slc39a*; *Zip* genes) of this metal (Reviewed by [2]). Recent studies also reveal that zinc can be stored and released from intracellular vesicular compartments and can function as a novel intracellular second messenger [3], [4]. For example, zinc appears to be essential for lipopolysaccharide induced signal transduction in monocytes and to influence IFN-γ expression in activated T-cells, and in mast cells labile zinc plays a role in regulation of caspase activation and NF-κB translocation (Reviewed by [5]). In the brain, synaptically released zinc triggers a metabotropic signal [6]. Zinc can modulate the proliferation and differentiation of mammalian cells by affecting several growth factor signaling cascades [7]. The mechanism by which zinc exerts these effects is an area of active investigation. Zinc can affect signal recognition, second messenger metabolism, protein kinase and protein phosphatase activities and transcription factor activity [5], [8]–[10].

Given the diversity of functions of zinc it is perhaps not surprising that zinc transporters have recently been found to play roles in carcinogenesis [2], [10], [11], as well as in embryonic development (Reviewed by [12]). For example, ZIP1, ZIP4, ZIP6, ZIP7 and ZIP10, members of the **Z**rt-**I**rt-like **p**rotein (*Slc39a*) superfamily [2], have each been implicated in specific cancers. ZIP1 is repressed in prostate cancer [11]. Aberrant accumulation of ZIP4 has been reported to contribute to the progression and aggressiveness of pancreatic cancer [13]–[16]. ZIP6 and ZIP7 may play roles in metastasis of estrogen receptor-positive breast cancer [10], [17] as well as in the proliferation, migration and invasion of cervical cancer [18]. Similarly, ZIP10 has been shown to affect the invasive behavior of breast cancer cells [19]. With regard to embryonic development, mutations in human ZIP4 cause the lethal genetic disorder of zinc metabolism acrodermatitis enteropathica, and the mouse *Zip4* gene is essential for proper early morphogenesis of the mouse embryo [20], [21]. To date *Zip4* is the only mammalian ZIP superfamily member which has been shown to be an essential gene; one that plays a fundamental role in adaptation to zinc deficiency. However, the *fear-of-intimacy* gene in *Drosophila* encodes a ZIP6-like zinc transporter that is essential during early development and plays a role in migration of germ cells [22]. Similarly, in zebrafish, a member of the ZIP superfamily (*zLIV-1*) is essential during early morphogenesis and plays a role in the epithelial-mesenchymal transition (EMT) during gastrulation [23].

Given the fundamental role of ZIP4 in mammalian development and adaptation to zinc deficiency, and the recent report of its role in pancreatic cancer, we examined whether this gene may play a fundamental role in carcinogenesis. Herein, we report that the *Zip4* gene is also actively expressed in hepatocytes in human and mouse hepatocellular carcinomas (HCC), and meta-analyses of the Geo and Oncomine databases further suggested that enhanced expression of the *Zip4* gene may occur in many different cancers. We present evidence that ZIP4 can repress apoptosis and enhance cell-cycle and cell migration. Therefore, ZIP4 can exert profound effects on cancer growth and metastasis.

## Results

### The Zip4 Zinc Transporter Gene is Activated in Human and Mouse Hepatocellular Carcinomas and May Also be Activated in Many Different Cancers

**Figure 1:**
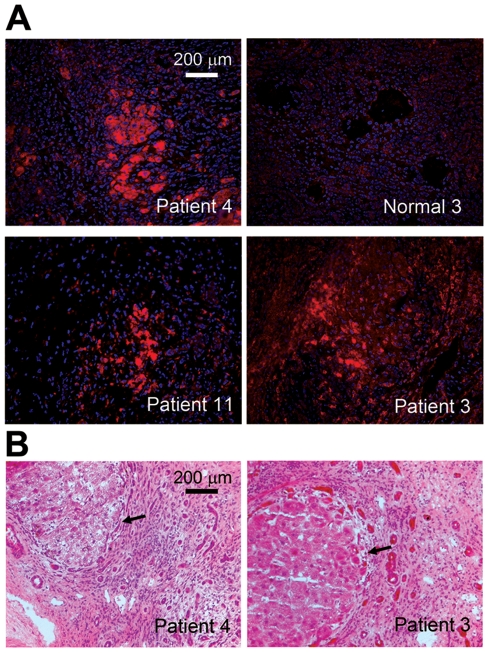
Immunofluorescence detection of ZIP4 in human HCC. **Part A:** Frozen sections of HCC from patients 3, 4 and 11 and normal liver tissue from patient 3 (see Table 1) were fixed, permeablized and blocked then ZIP4 was detected using an antipeptide antibody against mouse ZIP4 [26], [47]. The peptide immunogen is well conserved between mouse and human and specificity of this antibody has been established [26], [27], [46]. Sections were washed and incubated sequentially with biotinylated secondary antibody then QDot 655 streptavidin conjugate. Red indicates the presence of ZIP4 whereas blue indicates nuclei stained with DAPI. Adobe Photoshop image capture software was used to capture these images. **Part B:** Frozen serial sections from patients 3 & 4 stained with hematoxylin and eosin to reveal the cellular structure of the ZIP4 positive tumors. **Arrows** point to the boundary of well-circumscribed lesions containing ZIP4 positive hepatocytes.

We examined the expression of *Zip4* in human hepatocellular carcinomas (HCC). RNA extracted from a panel of biopsies of human HCC and surrounding control tissue was analyzed by real-time quantitative RT-PCR for *Zip4* mRNA and values were normalized to *GAPDH* mRNA (Table 1). Of the cancers examined, 16 of 23 had elevated levels (>2-fold) of *Zip4* mRNA compared with surrounding control tissue and in several of the HCC samples *Zip4* mRNA was dramatically elevated (10- to 77-fold). Similar results were obtained by normalizing *Zip4* mRNA values with those of another internal control *HPRT1* mRNA values (data not shown). The presence of ZIP4 protein in human HCC was confirmed by immunofluorescence detection in frozen sections (Fig. 1A). Cancerous tissues stained intensely for ZIP4 whereas surrounding cells and normal liver did not (Fig. 1A; see normal liver tissue 3 and Patient 3). Many hepatoadenoma/carcinoma cells within tumor tissues stained very intensely for ZIP4 (Fig. 1A; see top left panel, Patient 4). ZIP4 was also detected in hepatocytes from several of the samples which did not appear to have elevated *Zip4* mRNA (see Fig. 1; patient 11). Histological examination of liver sections from patients 3 and 4 (Fig. 1B) confirmed that ZIP4 positive cells were within well-circumscribed lesions which primarily contained hepatoadenoma/carcinoma cells, although we cannot exclude the possibility that some other cell-types may also express *Zip4* in these tumors.

**Table 1:** Aberrant Expression of *Zip4* (*Slc39a4*) in Human HCC.

	*Zip4* mRNA (x 10^-2^)*		Fold Increase
Patient No.	(Normal)	(Tumor)	(Tumor/Normal)
1	2.07	22.04	10.65
2	0.37	5.92	16.00
3	0.95	11.83	12.45
4	0.70	17.22	24.60
5	5.71	7.38	1.29
6	1.93	1.74	0.90
7	0.35	1.53	4.37
8	2.40	4.79	1.99
9	0.86	4.90	5.70
10	6.07	15.34	2.53
11	1.65	1.24	0.75
12	0.86	10.91	12.68
13	1.22	9.98	8.18
14	0.60	46.23	77.05
15	0.51	2.45	4.80
16	0.77	1.43	1.85
17	2.07	0.79	0.38
18	1.38	7.99	5.79
19	0.91	2.51	2.76
20	0.25	2.23	8.92
21	1.06	1.06	1.00
22	0.45	1.03	2.28
23	0.08	1.18	14.75

*Relative *Zip4* mRNA levels were normalized to that of *GAPDH* and are presented as 2∧(Ct*GAPDH*-Ct*ZIP4*).

Expression of *Zip4* in a mouse model of HCC was also examined, using *FXR*-knockout mice. FXR is a nuclear receptor essential for maintaining bile acid homeostasis. The *FXR*-knockout mice show increased bile acids and liver injury [24]. FXR deficiency in mice results in spontaneous liver carcinogenesis with hepatic adenoma, adenocarcinoma, and cholangiocyte carcinoma when reaching 8 to 10 months of age in both genders [24], [25]. In *FXR*-knockout mice older than 12 months, the incidence of liver tumor is 100% with the majority of the tumors being hepatocellular adenoma followed by hepatocellular carcinoma. The hepatocellular adenomas are characterized as well-circumscribed lesions with well-differentiated hepatocytes, whereas the hepatocellular carcinoma consist of both hepatocellular and cholangiocellular components.

**Figure 2:**
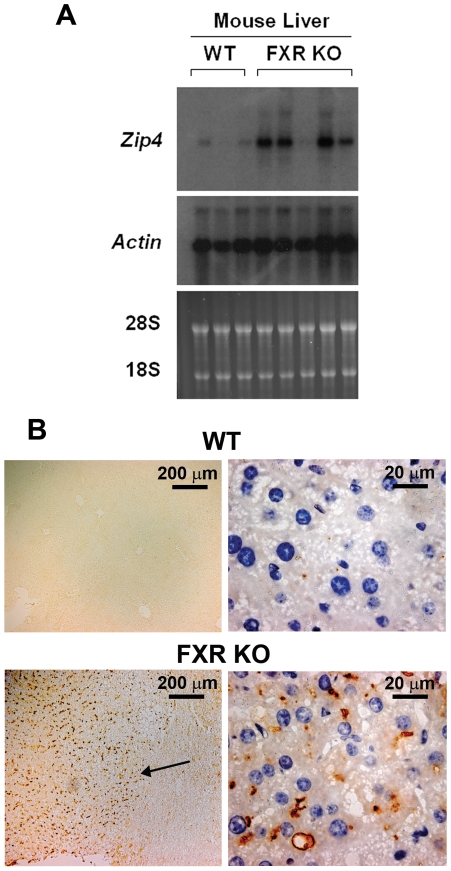
Detection of *Zip4* mRNA and ZIP4 protein in HCC from *FXR*-knockout and wild-type mice. **Part A:** HCC from *FXR*-knockout mice (**FXR KO**; 15 months of age) and normal liver from wild-type (**WT**) litter mates were analyzed by Northern blot hybridization to detect *Zip4* mRNA in total RNA. *Actin* mRNA served as a loading control in the Northern blots and staining of 28S and 18S rRNA served as a control for integrity and quantity of RNA loaded. **Part B:** Immunohistochemical detection of ZIP4 in paraffin sections of HCC from *FXR*-knockout and wild-type mice. **Arrow** is pointing to the boundary of a well-circumscribed lesion containing ZIP4-positive hepatocytes in the *FXR*-knockout liver. Brown deposits indicate ZIP4 staining. Blue color indicates hematoxylin stained nuclei.

Northern blot hybridization of RNA from normal liver and tumors from these mice (15 months old) revealed high levels of *Zip4* mRNA in 4 out of 5 of the tumor samples and the abundance of this mRNA in those tumors was similar to that found in mouse Hepa cells. In contrast, *Zip4* mRNA was rare in the normal liver samples (Fig. 2A) as reported previously [26]. The presence of ZIP4 protein in mouse HCC was confirmed by immunohistochemical detection in paraffin sections (Fig. 2B). Groups of cells staining intensely for ZIP4 were readily detected in all 5 tumor samples (one is shown in Fig. 2B), but no ZIP4 staining was detected in sections of normal liver (Fig. 2B; top panels) or in the cells surrounding the tumors in *FXR*-knockout mice (Fig. 2B; bottom panels). ZIP4 localization was restricted to the plasma membrane of hepatocytes within well-circumscribed lesions suggesting that these were hepatocellular adenomas (Fig. 2B). The majority of hepatocytes within these lesions displayed ZIP4 localization restricted to what appears to be the apical surface juxtaposed to a lumen, suggesting that these are well differentiated hepatocytes (Fig. 2B).

The finding that *Zip4* is aberrantly expressed in human and mouse HCC, as well as in pancreatic adenocarcinoma, suggested that *Zip4* expression might also be increased in other carcinomas. A meta-analysis of microarray data deposited in the Geo and Oncomine databases for *Zip4* (*Slc39a4*) expression supported this hypothesis (Table 2). In the Geo database many *Slc39a4* data sets did not reveal significant differences between normal and cancer samples, but evidence suggested elevated *Zip4* mRNA is found in some lymphoma, melanoma and metastatic colon cancer data sets and in data sets from two mouse models of HCC (*Mdr2* knockout and *Trnip* transgenic). In the Oncomine database *Zip4* was found to be among the top 10% of genes expressed in many human cancer types and was often associated with an aggressive stage of the cancer. Expression in human HCC was also confirmed. This meta-analysis suggests, but does not prove, that active expression of *Zip4* may occur in many different carcinomas. However, these analyses did confirm and extend our independent finding of active *Zip4* expression in HCC from humans and mice.

**Table 2:** Increased Expression of *Zip4* (*Slc39a4*) May also Occur in Many Different Cancers.

Database	Tumor type	Descriptor
Geo*	Diffuse large B-cell lymphoma	Mouse *Brd2* transgenic
	Metastatic colon cancer	Human
	Melanoma	Human
	HCC	Mouse *Trnip* transgenic
	HCC	Mouse *Mdr2* knockout
Oncomine**	Breast	Estrogen receptor positive
(Human)	Breast	Grade 3
	Colon	(see Bittner data set)
	Colorectal	(see Bittner data set)
	Gastric	(see Bittner data set)
	Ovarian	(see Bittner data set)
	Peritoneal	(see Bittner data set)
	Cervical	poorly differentiated
	HCC	Grade 4; poorly differentiated
	Lung	Grade 4
	Prostate	Grade 8–10; Grade 4
	Esophagus	Carcinoma

*Zip4 mRNA elevated in microarray compared with control. http://www.ncbi.nlm.nih.gov/geo.

**Top ten percent of genes expressed in a truncated listing of tumors as described/listed.

### Expression of the Zip4 Gene Reduces Apoptosis, Modestly Enhances Reentry into the Cell Cycle after G_0_/G_1_ Block When Zinc is Deficient, and Enhances Cell Migration

**Figure 3:**
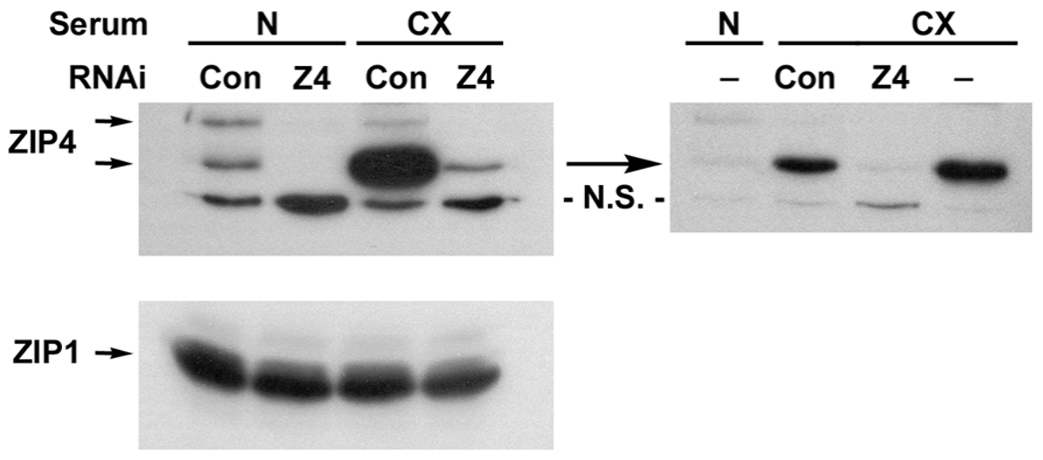
Western blot analysis of RNAi knockdown of *Zip4* in mouse Hepa cells. RNAi (two RNAi sequences) against mouse *Zip4* (**Z4**) and a scrambled RNAi control (**Con**) were expressed from the U6 promoter in lentivirus vectors that also express GFP driven by the *PGK* promoter. Cells were infected and then purified for GFP expression by FACS. Lentivirus infected cells and uninfected Hepa cells (**-**) were then cultured for 24 hr in zinc-adequate medium (**N**) which contains normal FBS or zinc-deficient medium (**CX**) which contains Chelex-treated FBS. Membrane preparations from these cells were then fractionated by SDS-PAGE, transferred to a membrane, and ZIP4 was detected using an antipeptide antibody against mouse ZIP4. A prominent ∼73 kDa ZIP4 band was detected (predicted size) as well as a larger ZIP4 aggregate. ZIP1 was detected as a loading control (bottom panel). In Hepa cells a non-specific (**NS**) band, which is predominately cytosolic, was detected in these membrane preparations.

To examine the functions of ZIP4 in HCC, we determined the effects of *Zip4* knockdown on apoptosis and progression through the cell cycle in mouse Hepa cells. These cells actively express the *Zip4* gene and regulate the stability of this mRNA and the endocytosis and processing or turn-over of this protein in response to zinc [27], [28]. ZIP4 protein is present at a low level in Hepa cells under zinc-adequate culture conditions but accumulates during zinc-deficiency (Fig. 3). Mouse Hepa cells were infected with lentivirus vectors which express two RNAi sequences against mouse *Zip4* mRNA or a scrambled control RNAi, and infected cells were purified by FACS using GFP expression from the viral vector. Western blot analysis of membrane proteins from these cells revealed that *Zip4* RNAi effectively reduced ZIP4 protein levels and largely prevented induction in response to zinc-deficient culture conditions, but had no effect on ZIP1 abundance (Fig. 3). The control RNAi did not alter ZIP4 abundance or effect induction of this protein during zinc-deficiency (Fig. 3).

**Figure 4:**
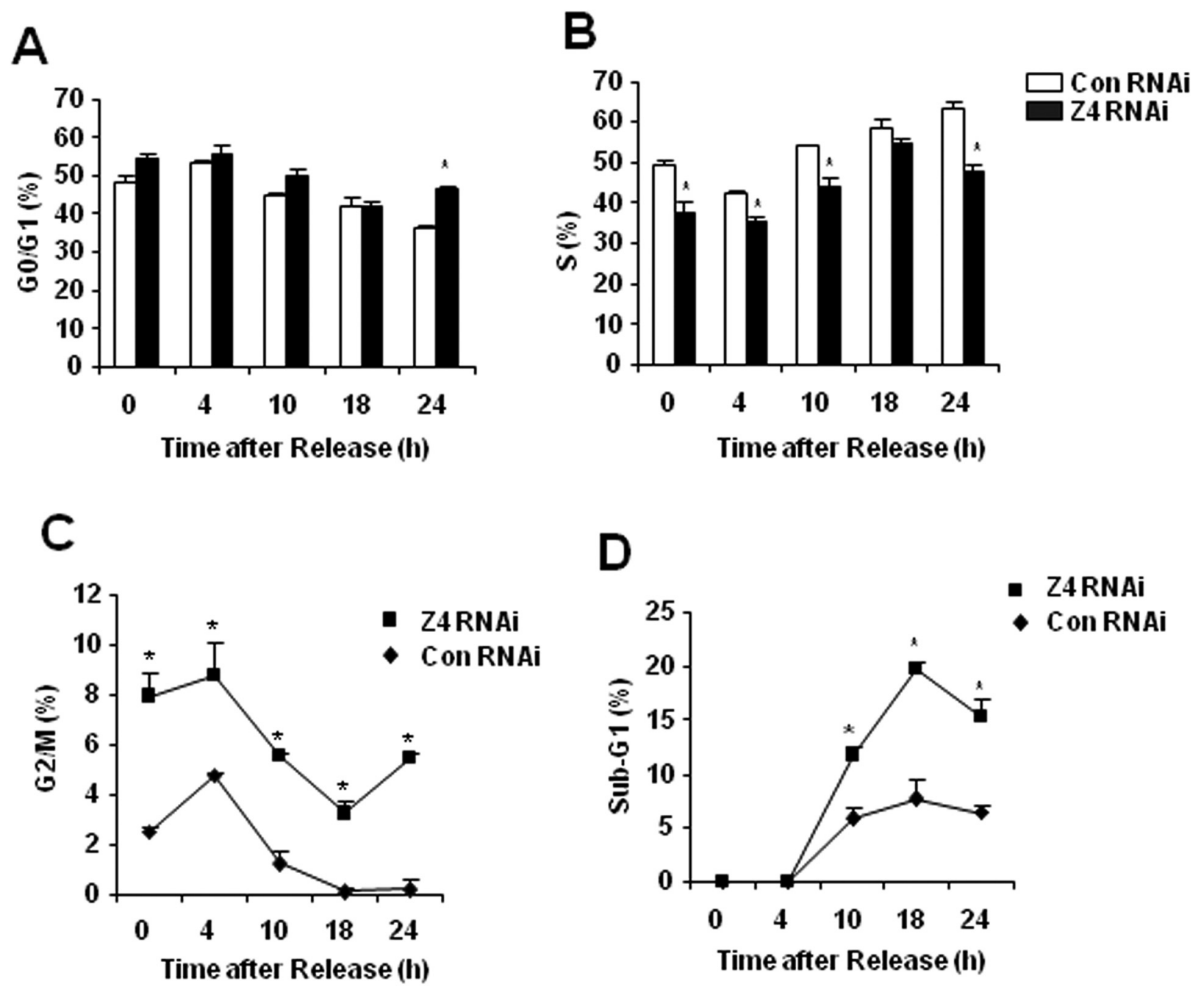
FACS analysis of the effects of *Zip4* knockdown on Hepa cell apoptosis and reentry into the cell cycle after release from hydroxyurea into zinc-deficient medium. Hepa cells expressing *Zip4* RNAi (**Z4 RNAi**) or control RNAi (**Con RNAi**) from lentivirus vectors were purified for GFP expression by FACS. Confluent cultures were replated at low density (1∶10) in zinc-deficient medium (DMEM plus 10% Chelex-treated FBS), and blocked overnight in G_0_/G_1_ using hydroxyurea. The medium was replaced with fresh zinc-deficient medium and progression through the cell cycle was quantified at the indicated times after release. Cell cycle parameters were monitored by propidium iodide staining followed by FACS. The percentage of cells in each phase of the cell cycle is presented within each FACS scan. **Part A:** Cells in G0/G1. **Part B:** Cells in S phase. **Part C:** Cells in G2/M. **Part D:** Cells in the sub-G_1_ fraction which is indicative of apoptotic cells. Data are expressed as the mean ± S.D. *Indicates a significant difference between *Zip4* RNAi and control RNAi (P<0.001).

*Zip4* RNAi and control RNAi Hepa cells, purified by flow sorting, were seeded at low density to facilitate a rapid onset of zinc deficiency, blocked using hydroxyurea to increase the number of cells in G_0_/G_1_, and then released from the block to reinitiate the cell cycle in zinc-deficient medium (Fig. 4). The number of cells in the various parts of the cell cycle was monitored by FACS at various times up to 24 hr (Table 3). After release from the hydroxyurea block into zinc-adequate medium, *Zip4* RNAi and control RNAi Hepa cells both progressed through the cell cycle at essentially indistinguishable rates (data not shown). In contrast, when released into zinc-deficient medium, cells expressing *Zip4* RNAi reentered the cell cycle more slowly than did those expressing control RNAi. At every time point examined the percentage of cells in S phase after release from the block was lower in the *Zip4* RNAi cells compared with the control RNAi cells (Fig. 4B) while the percentage of cells in G2 was greater in the *Zip4* RNAi cells compared with the control RNAi cells (Fig. 4B & C and Table 3). These data suggest that *Zip4* expression enhances cell cycle in Hepa cells. In addition to these findings, cultures expressing *Zip4* RNAi displayed a 2 to 3-fold increase in the sub-G_1_ fraction, indicative of apoptotic cells with fragmented nuclear DNA [29], after release into zinc-deficient medium (Fig. 4D and Table 3). In contrast to these results, expression of *Zip4* RNAi or control RNAi did not affect reentry into the cell cycle after a G_2_/M block using nocodazole, regardless of whether cells were released into zinc-adequate or zinc-deficient medium (data not shown).

**Table 3:** FACS Analysis of the Effects of ZIP4 Knockdown on Hepa cell Apoptosis and Progression after Release from Hydroxyurea Block into Zinc-Deficient Medium*.

Time after Release	Cell Cycle Phase	Con RNAi (Mean%)	(S.D.)	Zip4 RNAi (Mean%)	(S.D.)
0 h	SubG1	0	0	0	0
	G0/G1	48.27	1.47	54.255	1.23
	S	49.21	1.30	37.855	2.17
	G2/M	2.51	0.17	7.89	0.93
4 h	SubG1	0	0	0	0
	G0/G1	52.85	0.62	55.85	2.43
	S	42.41	0.78	35.34	1.15
	G2/M	4.73	0.15	8.80	1.28
10 h	SubG1	5.96	0.92	11.7	0.72
	G0/G1	44.90	0.95	50.31	1.55
	S	53.89	0.45	44.185	1.47
	G2/M	1.21	0.49	5.505	0.07
18 h	SubG1	7.77	1.64	19.72	0.56
	G0/G1	41.93	2.24	42.01	1.10
	S	57.98	2.35	54.785	0.60
	G2/M	0.08	0.11	3.205	0.50
24 h	SubG1	6.38	0.60	15.35	1.37
	G0/G1	36.08	1.08	46.07	0.69
	S	63.68	0.74	47.49	1.93
	G2/M	0.23	0.33	5.44	0.17

*Details of this experiment are presented in the legend to Figure 4 and in Materials and Methods.

**Figure 5:**
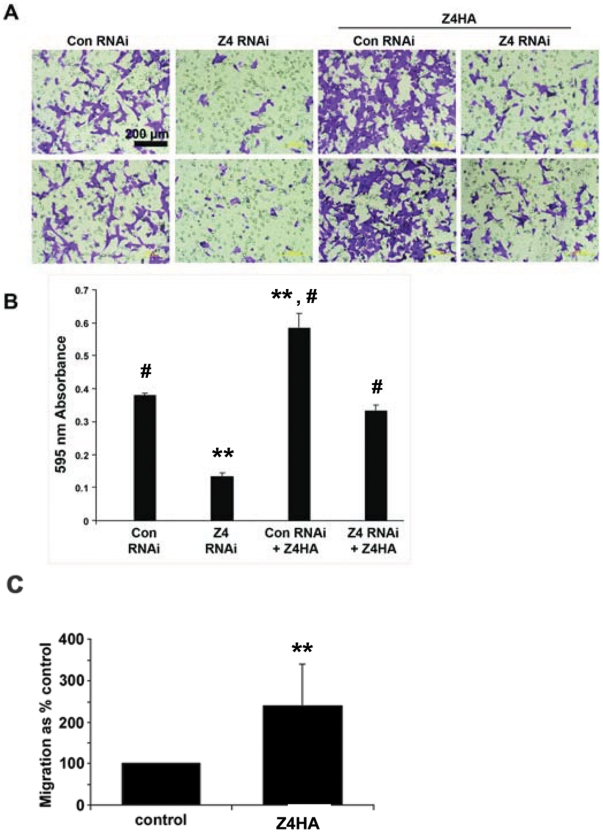
Effects of RNAi knockdown of *Zip4* and/or forced expression of *Zip4* on the migration of Hepa and MCF-7 cells. Hepa cells expressing *Zip4* RNAi (**Z4 RNAi**) or control RNAi (**Con RNAi**) from lentivirus vectors were purified for GFP expression by FACS. Cells were seeded onto fibronectin-coated Transwell inserts and allowed to migrate for a period of 16 h. Non-migratory cells were removed and cells that had migrated through the membrane were fixed and stained with crystal violet. Cell migration was visualized by microscopy (**Part A**) and quantified by measuring the absorbance of cell lysates at 595 nm (**Part B**). In parallel, Hepa cells expressing *Zip4* RNAi or control RNAi were transfected with a human *Zip4*-HA expression vector (**Z4HA**), serum starved overnight and then seeded onto Transwell inserts and allowed to migrate for 16 h. Migratory cells were visualized and quantified as described above. ** Indicates a significant difference from control RNAi and/or *Z4* RNAi, left two bars (P<0.001) and # indicates a significant difference from control RNAi and/or *Z4* RNAi/*Z4*HA (P<0.001). **Part C:** MCF-7 cells were transfected with an expression vector for mouse *Zip4*-HA [33] (**Z4HA**), or exposed to transfection reagent without vector (**control**), and then seeded onto fibronectin-coated Transwell inserts. Cells were allowed to migrate for a period of 16 h, non-migratory cells were removed and cells that had migrated through the membrane were fixed and stained. Cell migration was quantified by counting the numbers of cells in 5 randomly-chosen fields of view per membrane (n≥3 for each treatment) and results are plotted as the mean cell migration (percent of control ± S.D.). ** Indicates a significant difference between control and *Z4*HA transfected MCF-7 cells (P<0.001).

The potential functions of ZIP4 in hepatoma cells was investigated using Hepa cells that express control RNAi or *Zip4* RNAi, as well as these cells transiently transfected with a human *Zip4* cDNA expression vector encoding human ZIP4 with a carboxyl-terminal HA tag (Fig. 5A & B). Human *Zip4* mRNA would not be targeted efficiently by the RNAi against mouse *Zip4*. Forced expression of *Zip4* has been recently reported to increase cell proliferation, tumor volume and metastasis of pancreatic cells *in vivo* [13]. Herein, the ability of Hepa cells to migrate through a fibronectin-coated membrane was monitored. Hepa cells expressing control RNAi migrated through membranes more efficiently than did those expressing *Zip4* RNAi (Fig. 5A &B). Forced expression of human *Zip4*-HA in Hepa cells enhanced migration. Expression of human *Zip4*-HA in Hepa cells which also express *Zip4* RNAi against mouse *Zip4* restored migration activity, whereas it further augmented migration activity of Hepa cells expressing control RNAi. In parallel experiments we employed a well-established system using MCF-7 breast cancer cells which display a low background of cell migration *in vitro* [30]. In these cells, expression of *Zip7* has been shown to enhance migration [31] and *Zip4* is not expressed at significant levels [32]. Herein, MCF-7 cells were transiently transfected with an expression vector for mouse *Zip4* with a carboxyl-terminal HA tag [33]. The results show that forced expression of mouse *Zip4* also increased the migration of MCF-7 cells under these experimental conditions (Fig. 5C). These transfection experiments underestimate the effects of forced expression of mouse or human *Zip4*-HA since all of the cells were not transfected in these cultures. Taken together the above results demonstrate that ZIP4 can enhance cell migration.

## Discussion

Studies reported herein demonstrate that the essential zinc transporter ZIP4 is actively expressed in hepatocellular carcinoma (HCC) and may well be expressed in many other types of cancer. HCC is the fifth most common cancer in the world and third leading cause of cancer deaths resulting in over 600,000 deaths globally per year. The 5 year survival rate is reported to be below 5% (Reviewed by [34]). Thus, the finding of constitutively high levels of *Zip4* mRNA and protein in HCC, as well as in many cancers in humans, is intriguing and may represent a potential therapeutic target. Taken together with the recent demonstration that ZIP4 is aberrantly expressed in pancreatic carcinoma [13], our finding that *Zip4* mRNA and protein also accumulate in HCC suggests that aberrant activation of the *Zip4* gene may play a fundamental role in tumorigenesis.

How expression of the *Zip4* gene is turned-on in cancer cells is unknown. Active *Zip4* gene expression is normally highly cell-type specific [26], although very low level expression in some cultured cells has been detected by RT-PCR [35]. In the normal mouse liver *Zip4* mRNA is rare and unresponsive to dietary zinc [26], [27]. In contrast, *Zip4* mRNA abundance and protein stability are both regulated by several zinc-dependent post-transcriptional mechanisms in cells that actively express this gene (enterocytes, visceral endoderm, Hepa cells) [36]. Both mRNA and protein abundance are repressed when zinc is replete, whereas accumulation of ZIP4 protein at the apical surface of enterocytes and visceral endoderm cells and accumulation of *Zip4* mRNA occurs when zinc is deficient. The finding that ZIP4 localizes to what appears to be the apical surface of hepatocytes in the liver tumors from *FXR*-knockout mice suggests that either this protein is not zinc-regulated in those cells or that these cells are zinc deficient. Several studies have reported that HCCs have significantly lower levels of zinc than the surrounding tissues (Reviewed by [34]) which is consistent with the latter hypothesis. Whether this may also be the case for other cancers which may express ZIP4 is not known. Aberrant expression of *Zip4* has been previously documented in pancreatic ductal carcinoma, but the localization of ZIP4 in those cancers was not reported [13].

Our studies revealed that when zinc is deficient ZIP4 functions to repress apoptosis and may enhance reentry of hepatoma cells into the cell cycle following release from a G_0_/G_1_ block. How ZIP4 exerts these functions can be deduced from the fact that ZIP4 is essential for the acquisition of dietary zinc in humans, and for the transfer of zinc into the post-implantation conceptus and for resistance to zinc deficiency in mice [20]. Therefore, ZIP4 likely serves a fundamental role to facilitate zinc uptake in some tumors. Pancreatic cancer cells that over-express ZIP4 have higher zinc levels than do normal pancreatic cells and display increased *in vitro* proliferation and growth in nude mice [13]. A relationship between zinc, apoptosis and cell proliferation has been known for over 15 years [37]–[39]. Zinc can regulate caspases and apoptosis in a cell-type and concentration dependent manner [11]. Zinc deficiency can increase DNA damage and alter the expression of genes involved in cell cycle, apoptosis and DNA damage response and repair [38], as well as cause increased apoptosis in developing rat embryos [40] and in the skin [41]. Increased zinc stimulates DNA synthesis in embryonic stem cells [42] and attenuates apoptosis in human hepatoma cells in culture [43]. Taken together these findings support the concept that ZIP4 facilitates zinc uptake which, in turn, enhances DNA replication and repair and reduces apoptosis. Consistent with this concept knockdown of *Zip4* using RNAi in pancreatic cancer cells inhibited their growth in nude mice [14]. Other zinc transporters may serve the function of zinc uptake in some cancers.

Forced expression of either human or mouse *Zip4* was found herein to enhance the *in vitro* migration of Hepa cells and MCF-7 cells, respectively. In contrast, repressed expression of *Zip4* attenuated *in vitro* migration of Hepa cells. These findings are consistent with recent reports that forced expression of *Zip4* in pancreatic cancer cells increases tumor volume and metastasis in nude mice [13], whereas knockdown of *Zip4* in pancreatic cancer cells is associated with decreased cell proliferation, migration and invasion [14]. Taken together these studies provide strong support for the concept that ZIP4 serves a fundamental role in cancer.

The molecular mechanisms by which ZIP4 and zinc control growth and migration of cancer cells likely involves multiple signal transduction cascades. The effects of zinc and zinc transporters on signal transduction cascades appear to be, in part, cell-type specific. For example, mutations in ZIP13 cause a rare form of Ehlers-Danlos syndrome, and result in abnormal development of connective tissues possibly due to changes in signaling through the bone morphogenic protein/TGFβ signaling cascade [44]. In HeLa cells *Zip6* over-expression enhances SNAIL, a repressor of E-cadherin [18] and in Zebrafish zLIV-1 (ZIP6-like) regulates SNAIL and enhances the epithelial-mesenchymal transition during development [23]. Recent studies of pancreatic cancer reported that ZIP4 activates the IL-6/Stat3 pathway via CREB, leading to increased expression of neuropilin-1, vascular endothelial growth factor, and matrix metalloproteases [15], [16]. Zinc has been shown to modulate FoxO signaling in human hepatoma cells [43] and in mouse embryonic stem cells zinc stimulates DNA synthesis through the PI3K/Akt, MAPKs and mTOR pathways [42]. We noted that zinc availability can control the abundance of β-catenin in two hepatoma cell lines [Weaver and Andrews, unpublished observation]. Thus, ZIP4 enhanced zinc uptake may affect signal recognition, second messenger metabolism, protein kinase and protein phosphatase activities and transcription factor activity (Reviewed by [5], [10]). Whether ZIP4 has functions in addition to zinc uptake activity remains to be determined and further studies are required to delineate the mechanisms by which ZIP4 and other ZIP family members can alter signal transduction cascades in cancer cells.

## Materials and Methods

### Ethics Statement

All experiments involving mice were conducted in accordance with the National Institutes of Health guidelines for the care and use of experimental animals and were approved by the Institutional Animal Care and Use Committee (ACUP number 2007-1698: approved 1-19-2010).

### Human HCC Specimens

HCC specimens were obtained through the LTCDS (Liver Tissue Cell Distribution Service) under the NIH Contract – #N01-DK-7-0004/HHSN267200700004C (Minneapolis, Minnesota) [45].

### Real-Time PCR Quantification of Zip4 mRNA in Human HCC

Total RNA was isolated from tumor and surrounding normal tissue by Trizol extraction. Real-time PCR was performed using total RNA and products were detected using the SYBR Green PCR master mix (appliedbiosystems.com). PCR included the following components: 100 nM of each primer, diluted cDNA templates and SYBR green PCR master mix. Samples were denatured at 95°C for 10 min and amplified for 40 cycles at 95°C for 15 sec and 60°C for 1 min. The melting-curve data were collected to confirm PCR specificity. Each cDNA sample was run in triplicate, and the corresponding no-reverse transcriptase (RT) mRNA sample was included as a negative control. The *GAPDH* primers were included in every sample to control for sample variation. *Zip4* mRNA product in each sample was normalized to that of the *GAPDH* mRNA. The amount of PCR products was measured by threshold cycle (Ct) values and relative mRNA levels are presented as unit values of 2̂[Ct(*GAPDH*) -Ct(*Zip4*)]. The primer sequences for human *Zip4* mRNA (*Slc39a4*) were: sense 5′ AAGCACTGCTGCTGAACCTGGCCT 3′; and antisense 5′ GATGTCATCCTCGTACAGGGACAGCAGC 3′. The primer sequences for the human *GAPDH* mRNA were: sense 5′ ACATCATCCCTGCCTCTACTGG 3′; and antisense 5′ TCCGACGCCTGCTTCACC 3′.

### Immunofluorescence and Immunohistochemical Detection of ZIP4, and Histology of Human HCC Tissue

Frozen sections of human HCC and normal liver were thawed directly into phosphate-buffered saline (PBS) containing freshly prepared 2% paraformaldehyde and 0.1% Triton-X-100 for 30 min at room temperature. Sections were washed with PBS and incubated in PBS containing 50 mM lysine for 30 min to quench autofluorescence. Sections were washed in PBS, incubated in PBS containing 0.1% Triton-X-100/0.1% Tween 20 for 20 min, blocked with 10% normal goat serum in PBS for 1 hr and ZIP4 was detected, as described previously, using an antipeptide antibody against mouse ZIP4 [26]. The peptide immunogen is well conserved between mouse and human and specificity of this antibody has been established [26], [27], [46]. Sections were washed thoroughly with PBS and incubated sequentially with biotinylated secondary antibody (1∶500; jacksonimmuno.com) and QDot 655 streptavidin conjugate (1∶500; invitrogen.com). Slides were mounted with glycerol containing 4′,6-diamidino-2-phenylindole (DAPI) to stain nuclei and visualized using a Leica DM 4000B (Leica-microsystems.com) with Adobe Photoshop image capture software (adobe.com). For histology of human HCC, frozen serial sections from tissues assayed by immunofluorescence (above) were thawed directly in PBS containing freshly prepared 4% paraformaldehyde, washed in PBS, stained with hematoxylin and eosin and photographed using a Leica DM 4000B, as above.

Immunohistochemistry was employed to detect ZIP4 in *FXR*-knockout mouse livers and normal liver using the Zymed Laboratories Inc. Histostain-SP kit (invitrogen.com) for rabbit primary antibody and diaminobenzidine substrate staining, as described previously [47]. Tissues were fixed overnight in 4% paraformaldehyde, embedded in paraffin, and sectioned. Sections were deparaffinized, and subjected to antigen retrieval in 10 mM citrate (pH 6.0) at 95°C for 10 min. Sections were then treated with 1% peroxide, blocked with 10% normal goat serum, and incubated with the primary antiserum (1∶600 dilution). Sections were washed with PBS, incubated with biotinylated secondary antibody followed by streptavidin-conjugated horseradish peroxidase and then developed in diaminobenzidine. Slides were then stained with hematoxylin and photographed using a Leica DM 4000B with Adobe Photoshop image capture software.

### A Farnesoid X Receptor (FXR)-Knockout Mouse Model of HCC

The *FXR*-knockout mice were generated and back-crossed into the C57BL/6 genetic background, as described previously [24]. The genotype was confirmed by a PCR-based genotyping protocol. Mice were allowed free access to water and food and were exposed to a 12 hr light/12 hr dark cycle. Livers were removed from 15-month old male mice and snap-frozen in liquid nitrogen or frozen in OCT-compound.

### Northern Blot Hybridization

Total RNA (3–6 µg) was isolated, size-fractionated by agarose-formaldehyde gel electrophoresis and transferred and UV cross-linked to nylon membranes, as described in detail previously [48], [49]. Northern blot membranes were hybridized and washed under stringent conditions. Hybrids were detected by autoradiography at −80°C. Duplicate gels were stained with acridine orange to ensure equivalent loading and integrity of total RNA. Probes for mouse *Zip4* and *actin* mRNAs were as described [47]. Probes were used at 2×10^6^ cpm/ml of hybridization solution.

### Zinc-Adequate and Zinc-Deficient Cell Culture Conditions

Mouse Hepa cells were maintained at 37°C in a humidified 5% CO_2_ incubator in Dulbecco's modified Eagle medium (DMEM) containing 10% heat-inactivated fetal bovine serum (FBS), 100 units penicillin/ml, and 100 µg streptomycin/ml. To generate zinc-deficient conditions in culture, heat-inactivated fetal bovine serum (FBS) was treated with Chelex-100 resin, as described [33]. Essential metals except zinc were repleted by the DMEM which was adjusted to 10% Chelex-treated FBS. Cells were plated at a density of 5×10^4^ cells per 10 cm plate in either zinc-adequate (normal FBS) or zinc-deficient (Chelex-treated FBS) medium.

### Zip4 RNAi Lentivirus Construction

*Zip4* RNAi and control RNAi lentiviral constructs were created in third generation lentiviral vectors, amplified in HEK293 cells, purified by centrifugation, and titered in the Washington University Core Facility as described [50]. The packaging genes were separated on two plasmids which lack both LTRs and have no viral packaging signal. The following viral genes were deleted from the packaging vector: *env*, *vpr*, *vpu*, *vif* and *nef*. This vector has a self-inactivating LTR and envelope, *VsVg*, is expressed on a separate plasmid [51]. The RNAi against mouse *Zip4* and the scrambled RNAi control were expressed from the U6 promoter; this vector also expressed green fluorescence protein (GFP) driven by the *PGK* promoter. The oligos listed below were annealed, phosphorylated, and ligated into this vector just downstream of the U6 promoter at the AarI site.

### Zip4 RNAi

#### Zip4sh468 duplex

5′ ACCGGGAGAAGACCTGTGTAGATCTCTTCCTGTCAAGATCTACACAGGTCTTCTCC 3′

3′ CCTCTTCTGGACACATCTAGAGAAGGACAGTTCTAGATGTGTCCAGAAGAGGAAAA 5′

#### Zip4sh1332 duplex

5′ ACCGGGACCAGGATTCTGAGAAAGACTTCCTGTCATCTTTCTCAGAATCCTGGTCC 3′

3′ CCTGGTCCTAAGACTCTTTCTGAAGGACAGTAGAAAGAGTCTTAGGACCAGGAAAA 5′

### Scrambled control RNAi

#### shScrTop

5′ ACCGGCGCCTAATTGTCTGGACTATCTTCCTGTCAATAGTCCAGACAATTAGGCGC 3′

#### shScrBottom

5′ AAAAGCGCCTAATTGTCTGGACTATTGACAGGAAGATAGTCCAGACAATTAGGCGC 3′

### Lentivirus Infection and Purification of Mouse Hepa Cells

Mouse Hepa cells were incubated with *Zip4* RNAi and control RNAi lentiviruses at a multiplicity of infection of 5∶1 overnight. Cells were washed extensively and then incubated for 24 hr in DMEM containing 10% FBS. A single cell suspension of these cells was prepared in PBS containing 20% FBS and analyzed by fluorescence activated cell sorting (FACS) for GFP expressed from the lentivirus construct. Fluorescence in the GFP channel was monitored and the intensity of autofluorescence in the wild-type cells was used to define the base line fluorescence. Cells with signal intensities clearly greater than that value were collected and used for experiments. At least 95% of the flow sorted cells were GFP positive.

### Western Blot Detection of ZIP4

Membrane proteins were prepared from Hepa cells and analyzed as described [27], [47]. Membrane proteins (20 to 25 µg) were resolved by 8% SDS-polyacrylamide gel electrophoresis (PAGE) and transferred to polyvinylidene difluoride membranes. Membranes were blocked overnight and then incubated with primary antibody in blocking solution at the appropriate dilution (ZIP4 antiserum 1∶800; ZIP1 antiserum 1∶800) for 2 hr at room temperature. After extensive washing, membranes were incubated with goat anti-rabbit horseradish peroxidase-conjugated secondary antibody and the blot was developed using ECL Plus reagent (amershambiosciences.com) according to manufacturer's instructions and detected using Kodak BioMax MS film (kodak.com).

### Cell Cycle Analysis by FACS

Hepa cells expressing *Zip4* RNAi or control RNAi were split 1∶10 from confluent cultures into zinc-adequate or zinc-deficient medium and then blocked in G_0_/G_1_ by incubating for 16 hr with 100 mM hydroxyurea (sigmaaldrich.com) or blocked in G_2_/M by incubating for 16 hr with 0.1 µg/ml nocodazole (sigmaaldrich.com). The blocking medium was then replaced with zinc-adequate or zinc-deficient medium to allow cells to progress in the cell cycle, and cells were harvested at different times thereafter. Cell cycle parameters were monitored by propidium iodide (PI) staining followed by FACS using an Epics XL Flow Cytometer (beckman.com) [52].

### ZIP4 and Cell Migration Assay

Hepa cells expressing *Zip4* RNAi or control RNAi were plated onto 60 mm dishes and transfected with 4 µg of a human *Zip4*-HA expression vector or pcDNA3.1 control vector using Lipofectamine 2000 (invitrogen.com) in antibiotic free media according to the manufacturer's instructions. After 6 h the cells were serum starved for 24 h and 5×10^4^cells were seeded onto Transwell inserts (8 µm pore size; Culterx 96 well cell migration assay: amsbio.com). Cells were allowed to migrate for 16 h after which the non-migratory cells were removed from inside the chamber with a cotton swab. Cells that had migrated through the membrane pores were fixed for 10 min (3.7% v/v formaldehyde in PBS), stained with 0.1% crystal violet for 15 min, washed with PBS, and photographed using a Microfire/Qcam CCD Olympus 1×81 microscope. Crystal violet stained cells were lysed in 1% SDS for 30 min and absorbance of the lysate was measured at 595 nm in a plate reader (BioTek, synergy HT: biotek.com). Results were plotted as the mean absorbance ± S.D.

MCF-7 cells were seeded at 2×10^5^/cm^2^ and grown until 80% confluent in phenol-red-free RPMI supplemented with 5% fetal calf serum, 200 mM L-glutamine, 10 IU/ml penicillin, 10 µg/ml streptomycin, and 2.5 µg/ml fungizone. Cells in 60 mm dishes were transfected with 8.8 µg of an expression vector for mouse *Zip4*-HA [33] and 27.5 µl of Lipofectamine 2000 (invitrogen.com) in antibiotic free media according to the manufacturer's instructions. As a control, cells were exposed to Lipofectamine 2000 without expression vector. After 6 hr the cells were counted and 2×10^4^ cells were seeded onto fibronectin-coated (1 µg/ml) Transwell inserts (8 µm pore size; bdbiosciences.com). Cells were allowed to migrate for a period of 16 h after which the non-migratory cells were removed from inside the chamber with a cotton swab. Cells that had migrated through the membrane pores were fixed (3.7% v/v formaldehyde in PBS) and stained with crystal violet. Cell migration was quantified by counting the numbers of cells in 5 randomly-chosen fields of view per membrane (n≥3 for each treatment) and results were plotted as the mean cell migration (percent of untreated cells ± S.D.).

### Meta-Analyses of Zip4 mRNA Microarray Data Deposited in the Geo and Oncomine Public Databases

The Geo microarray database at the NCBI was accessed at the following URL: http://www.ncbi.nlm.nih.gov/geo. This database was queried for Gene Profile of *Slc39a4* and cancer, at the time of submission of this manuscript, which yielded 106 hits; *Slc39a4* and hepatocellular carcinoma which yielded 5 hits; *Slc39a4* and lymphoma which yielded 21 hits, *Slc39a4* and colon cancer which yielded 5 hits; and *Slc39a4* and melanoma which yielded 18 hits. Among these data sets several cancers which showed increased expression of *Zip4* mRNA were noted. The Oncomine database was accessed at the following URL: http://www.oncomine.org. This database was queried for *Slc39a4* among the top ten percent of genes expressed in tumors.

### Statistical Analyses

Data were analyzed using the Students'-T-Test to determine P values. Data are expressed as the mean ± Standard Deviation (S.D.)
